# Flow diverter therapy for mirror-like aneurysms at the middle cerebral artery trifurcation: a case report

**DOI:** 10.3389/fsurg.2025.1718934

**Published:** 2026-01-08

**Authors:** Lin Zheng, Jinlu Yu

**Affiliations:** 1 Department of Neurosurgery, People’s Hospital of Tonghua, Tonghua, China; 2 Department of Neurosurgery, The First Hospital of Jilin University, Changchun, China

**Keywords:** middle cerebral artery, aneurysm, flow diverter, treatment, case report

## Abstract

Flow diverters (FDs) can be deployed to treat certain intracranial aneurysms by covering the parent artery origin without direct neck coverage, though this approach remains uncommon. This present case demonstrated successful treatment of mirror-like aneurysms at the middle cerebral artery (MCA) trifurcation using this technique. A 57-year-old male patient presented with subarachnoid hemorrhage. Computed tomography angiography revealed mirror-like aneurysms of the left MCA. Physical examination showed a Hunt-Hess scale score of Grade II. Endovascular treatment was performed. Under general anesthesia, digital subtraction angiography (DSA) demonstrated left MCA trunk division into triple branches. The superior and inferior branches displayed mirror-like dissecting aneurysms, while the middle branch appeared normal. Following coiling of both aneurysms, a FD was deployed in the middle branch to cover the origins of the superior and inferior branches. Postoperatively, the patient developed motor aphasia due to vasospasm. The continuous nimodipine infusion treatment resulted in the relief of the symptom. At six-month follow-up, the patient had mild motor aphasia. DSA confirmed occlusion of both the superior and inferior branches with the aneurysms, while preserving the middle branch to supply the left MCA territory. This case demonstrates that for mirror-like MCA aneurysms located at the superior and inferior branches of a MCA trifurcation, deploying a FD in the normal middle branch to cover the origins of the two aneurysmal branches may result in occlusion of the parent arteries harboring the aneurysms. However, this novel approach warrants cautious application as it remains non-routine.

## 1 Introduction

Since 2000, flow diverters (FDs) have revolutionized endovascular treatment (EVT) for intracranial aneurysms by reconstructing the parent artery, redirecting blood flow, and altering hemodynamic flow within the aneurysm (1). Even the FD had been deployed for middle cerebral artery (MCA) aneurysms (2). Standard FD deployment requires spanning the aneurysm neck to achieve optimal therapeutic outcomes. In select cases, however, an FD may be positioned across the parent artery origin rather than the aneurysm neck, inducing indirect flow diversion to attenuate hemodynamic stress and mitigate rupture risk (3). Of particular significance, FD deployment can also induce occlusion of the covered branch. This phenomenon presents a novel therapeutic paradigm for MCA mirror-like aneurysms, an approach that has not been previously reported. Such a valuable case was reported.

## 2 Case presentation

### 2.1 Patient's onset and diagnosis

**Figure 1:**
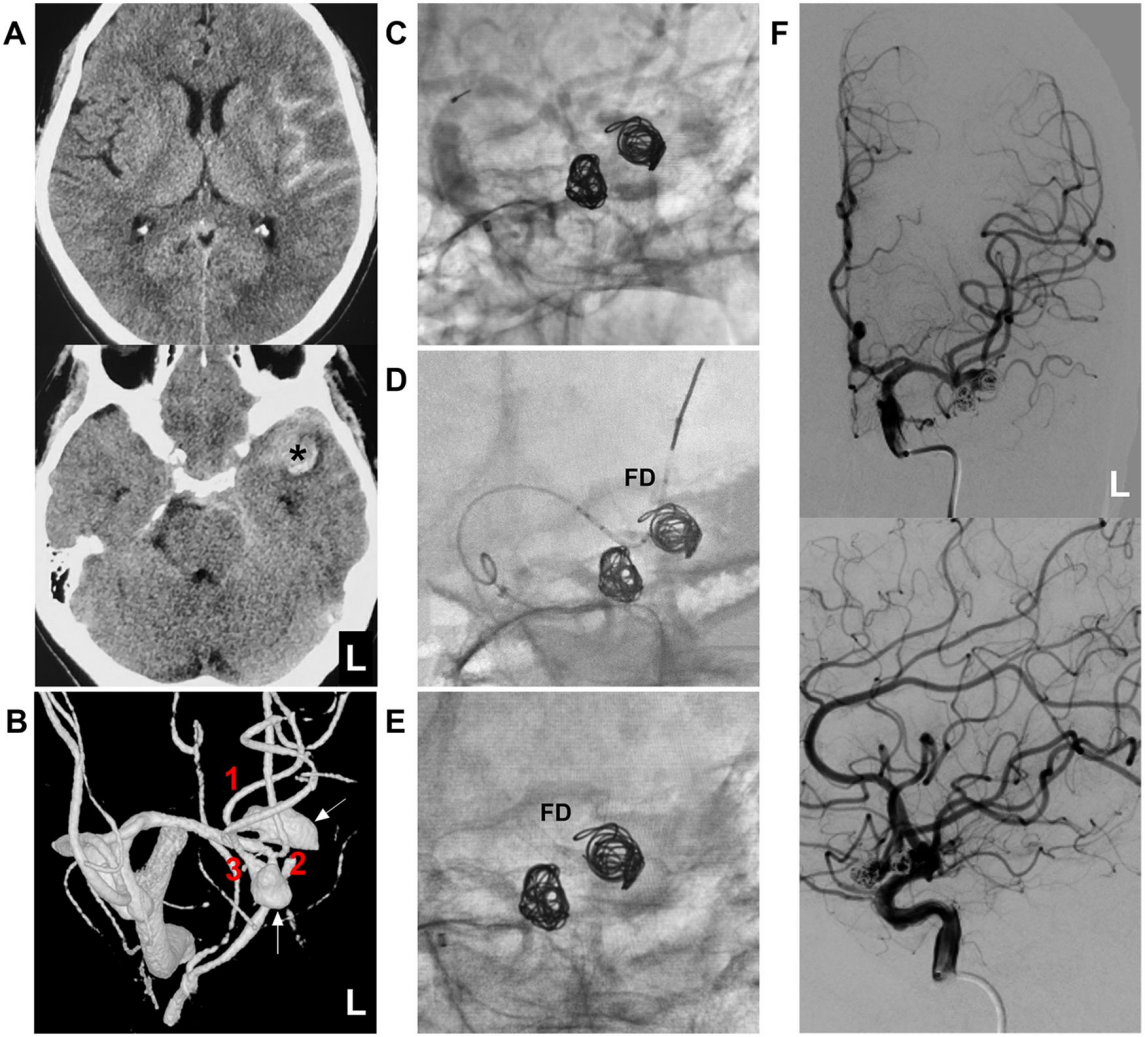
Preoperative CT and EVT course. **(A)** CT images demonstrate left subarachnoid hemorrhage (SAH) (upper panel) and a small left Sylvian fissure hematoma (asterisk, lower panel), consistent with aneurysm rupture. **(B)** Three-dimensional DSA reveals the left MCA trifurcation (branches 1-3), with two aneurysms (arrows) located near the origins of the superior and inferior MCA branches. These aneurysms exhibit mirror-like symmetry. **(C)** Unsubtracted DSA confirms incomplete coiling of the two aneurysms. **(D)** X-ray demonstrates selective delivery of the FD in the middle MCA branch. **(E)** X-ray confirms successful FD deployment in the middle MCA branch. **(F)** DSA images of the left internal carotid artery in lateral (upper panel) and anteroposterior (lower panel) views demonstrate the patency of all three MCA branches post-EVT. CT, computed tomography; DSA, digital subtraction angiography; EVT, endovascular treatment; FD, flow diverter; L, left; MCA, middle cerebral artery; SAH, subarachnoid hemorrhage.

A 57-year-old male patient was admitted to the hospital with a four-hour history of severe headache and vomiting. Computed tomography (CT) revealed left subarachnoid hemorrhage (SAH) and a small hematoma in the left Sylvian fissure (Figure 1A). Subsequent CT angiography (CTA) identified two symmetrical mirror-like aneurysms at the bifurcation of the left MCA. On physical examination, the patient was able to follow commands, exhibited neck stiffness, and demonstrated grade V muscle strength in all four limbs. The patient's condition was classified as Grade II on the Hunt-Hess scale.

The patient is of Han ethnicity. His medical history was noncontributory, with no prior history of stroke, systemic hypertension, or diabetes mellitus. There was also no family history of intracranial aneurysm or hemorrhage. All preoperative tests (laboratory and imaging) confirmed eligibility for general anesthesia and EVT. Three hours before the procedure, a loading dose of aspirin 300 mg and ticagrelor 180 mg was administered orally as dual antiplatelet therapy (DAPT). The EVT was subsequently performed.

### 2.2 Therapeutic process

Under general anesthesia, digital subtraction angiography (DSA) demonstrated that the MCA trunk trifurcated into superior, middle, and inferior branches. At the trifurcation site, there were two mirror-like dissecting aneurysms (8 mm and 7 mm in diameter, respectively) at the origins of the superior and inferior branches, while the middle branch appeared anatomically normal (Figure 1B). After an 8F guiding catheter was positioned in the petrous internal carotid artery, a 5F distal access catheter (Tongqiao Medical Technology Co., Ltd., China) was advanced into the cavernous internal carotid artery.

The Echelon-10 microcatheter (Medtronic, Irvine, CA, USA) was then advanced under the guidance via a Synchro-14 microguidewire (Stryker Neurovascular, Fremont, CA, USA) into the lower branch aneurysm, where an Axium 7 mm–30 cm coil (Medtronic, Irvine, CA, USA) was deployed for embolization. Subsequently, the microcatheter was navigated into the upper branch aneurysm, and an Axium 8 mm–30 cm coil was delivered to complete the embolization. The embolization of the two aneurysms was incomplete (Raymond–Roy grade III), and the parent arteries remained patent (Figure 1C).

Following embolization of the two aneurysms, the middle branch of the MCA was catheterized with an XT-27 microcatheter (Stryker Neurovascular, Fremont, CA, USA). A 2.6 mm–18 mm Lattice FD (AccuMedical, Beijing, China) was then deployed through this microcatheter to cover the origins of both the upper and lower branches of the MCA (Figures 1D,E). During FD deployment, tirofiban (4 mL by intravenous bolus) was administered. After completing the EVT, DSA confirmed the patency of all three MCA branches (Figure 1F). Post-EVT, tirofiban infusion was planned to be continued at 4 mL/h the following day. Starting on the second day, DAPT was to be transitioned to aspirin 100 mg once daily and ticagrelor 60 mg twice daily, to be maintained for 6 months post-EVT.

### 2.3 Postoperative and follow-up outcomes

**Figure 2:**
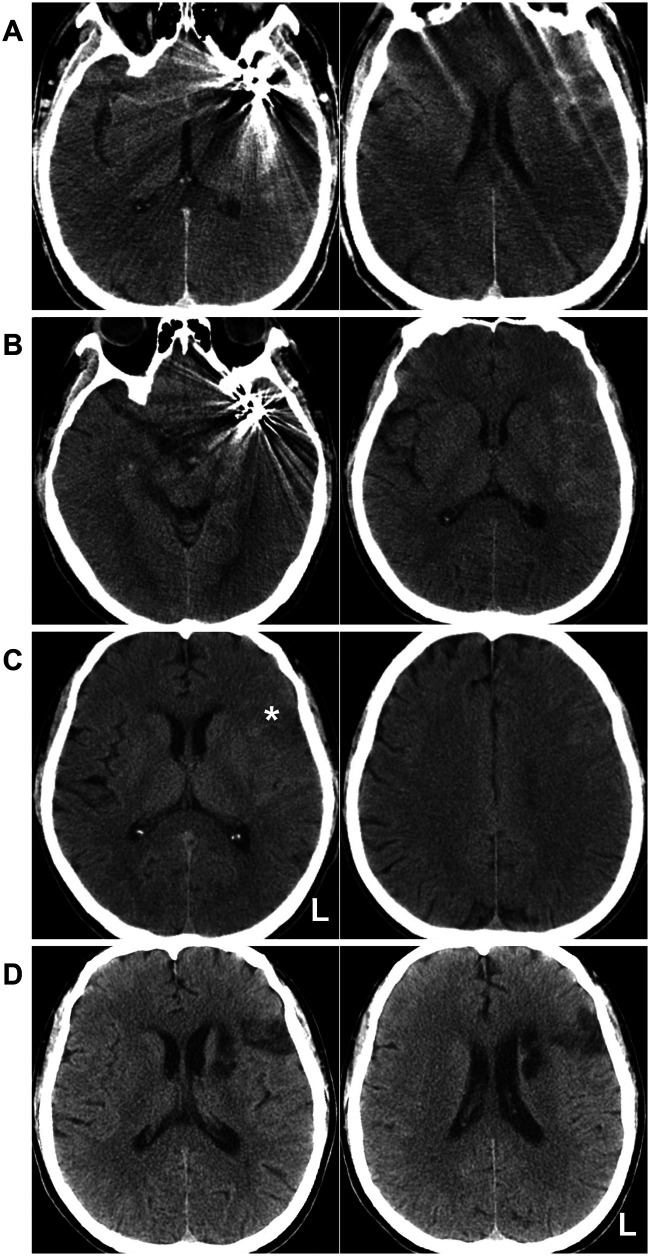
Postoperative and follow-up CT findings. **(A)** Postoperative day 1 CT images revealed no rebleeding or edema. **(B)** Postoperative day 7 CT demonstrated no apparent infarction, with absorption of subarachnoid hemorrhage. **(C)** Postoperative one-month CT showed an infarction (asterisk) in the left inferior frontal gyrus opercular region. **(D)** Nearly six-month follow-up CT revealed multiple infarcts in the left frontal lobe and head of the caudate nucleus. CT, computed tomography; L, left.

Postoperatively, the patient demonstrated no new neurologic deficits. Day 1 CT confirmed no rebleeding or edema (Figure 2A). His headache gradually resolved. However, by postoperative day 7, the patient developed motor aphasia. CT showed no rebleeding or obvious infarction (Figure 2B), prompting consideration of vasospasm. Patients received intravenous fluids (2,000 mL/24 h) and a continuous nimodipine infusion (4 mL/h) for 7 days, which resulted in marked improvement of motor aphasia. At one-month follow-up, the patient still had mild motor aphasia with otherwise normal function, while CT demonstrated a small infarction in the opercular part of the inferior frontal gyrus (Figure 2C).

**Figure 3:**
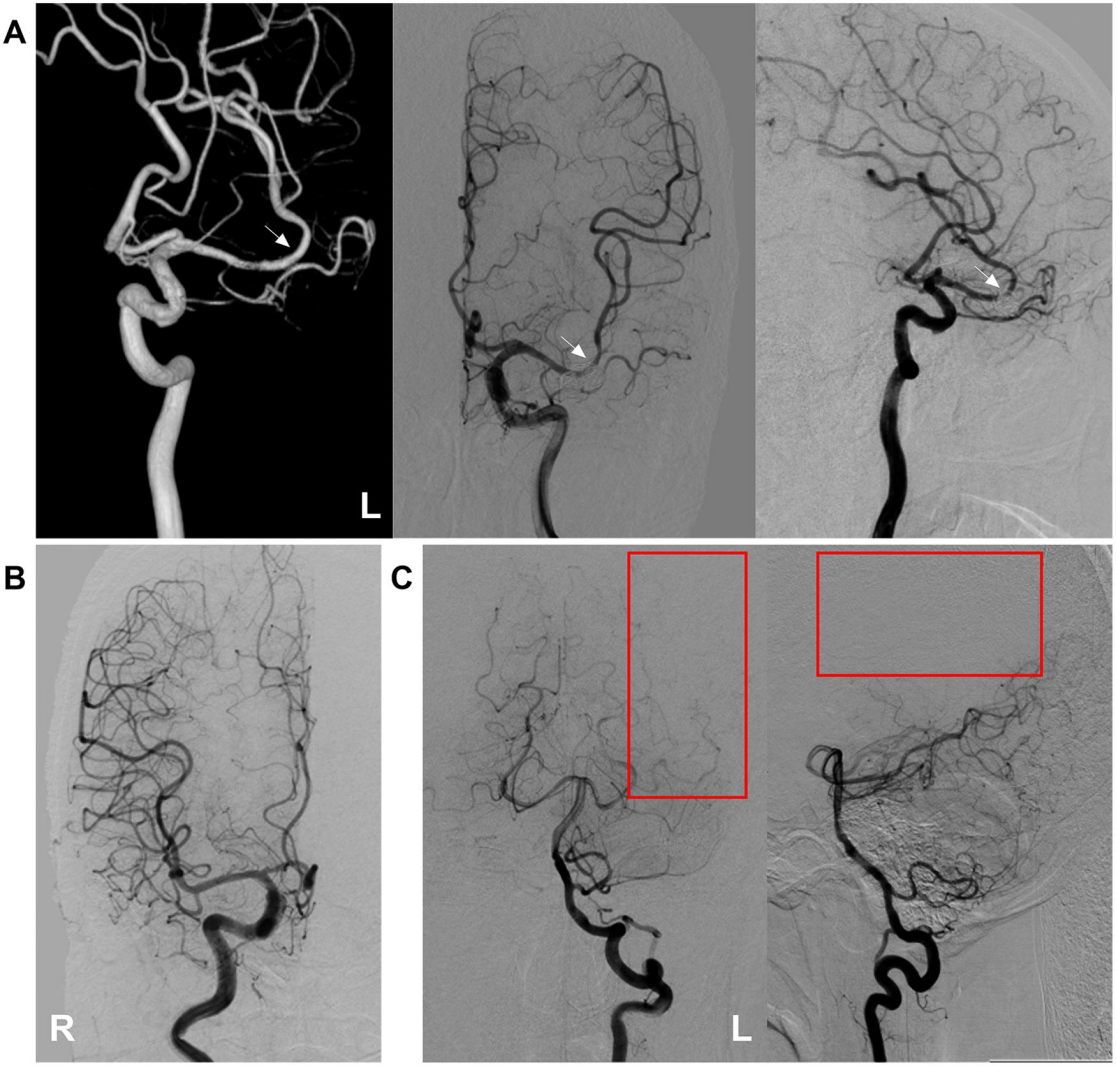
Follow-up DSA findings. **(A)** Nearly six-month follow-up angiographies of the left internal carotid artery at three-dimensional DSA (left panel) and DSA images in anteroposterior (middle panel) and lateral (right) views confirmed occlusion of both the upper and lower MCA branches (including the two aneurysms), while preserving the middle branch (arrows) to maintain perfusion of the MCA territory. **(B)** DSA of the right internal carotid artery showed no collateral blood supply to the left MCA region via the anterior cerebral artery. **(C)** DSA images of the vertebral artery in anteroposterior (middle panel) and lateral (right) views confirmed no collateral blood supply to the left MCA region via the posterior cerebral artery (red frames). DSA, digital subtraction angiography; L, left; MCA, middle cerebral artery; R, right.

**Figure 4:**
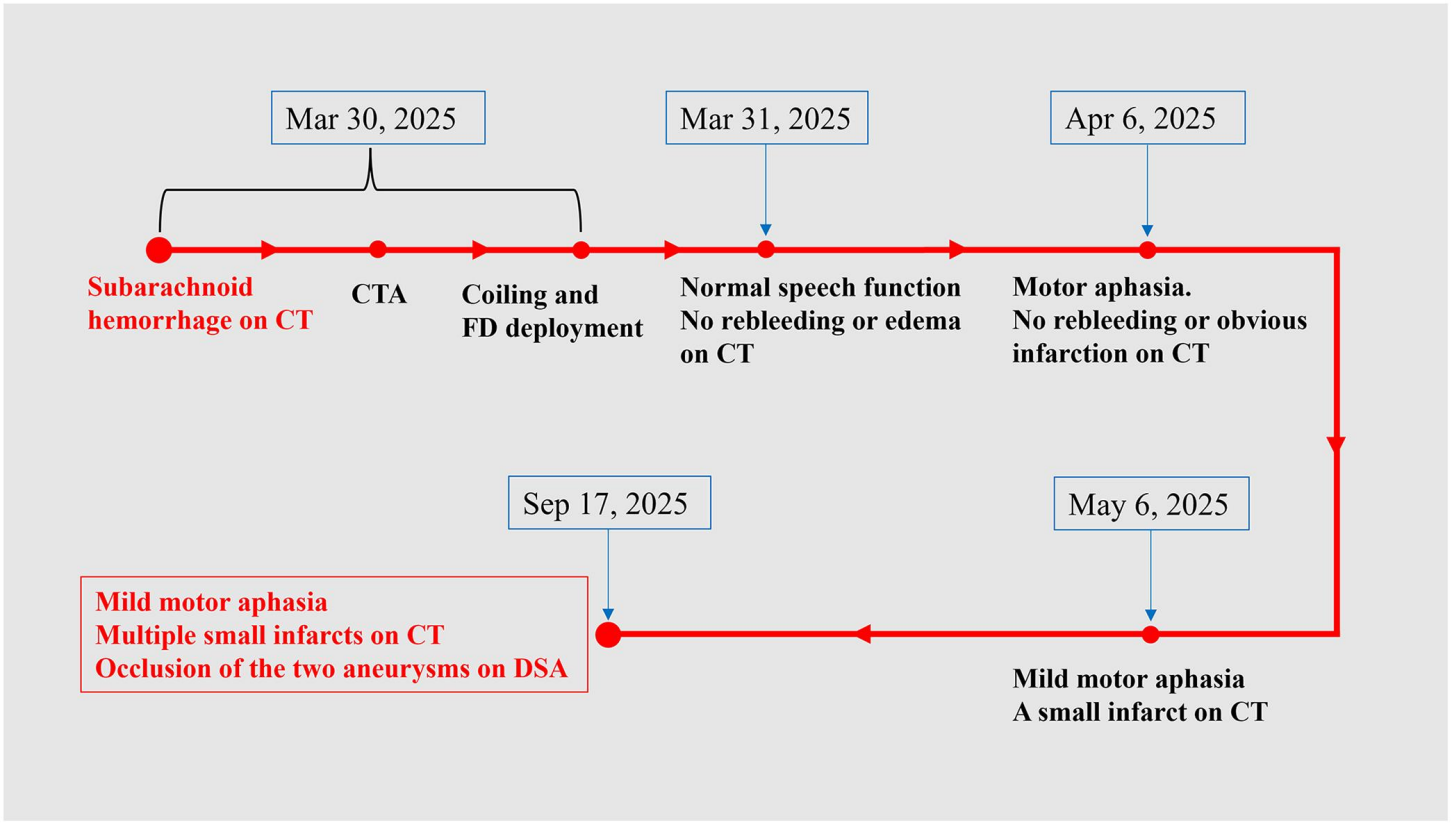
Patient timeline. CT, computed tomography; CTA, computed tomography angiography; DSA, digital subtraction angiography; FD, flow diverter.

At the nearly six-month follow-up, the patient continued to exhibit persistent mild motor aphasia. No additional neurological symptoms were observed. CT revealed multiple infarcts in the left frontal lobe and head of the caudate nucleus (Figure 2D). DSA confirmed occlusion of both the upper and lower branches of the MCA, with the two aneurysms. The middle branch remained patent to supply the MCA territory (Figure 3). Ticagrelor was discontinued, while aspirin was maintained. The timeline is shown in Figure 4.

## 3 Patient perspective

During the nearly six-month follow-up, the patient shared his experience: “Before the EVT, the headache was excruciatingly severe, and the diagnosis of a cerebral aneurysm led to a treatment plan I considered highly appropriate. Although I later experienced complications from cerebral vasospasm, which resulted in incomplete aphasia, by the sixth month, the recovery was quite satisfactory. What made me particularly happy was the follow-up imaging confirming complete healing of the aneurysm.”

## 4 Discussion

Traditionally, MCA aneurysms have been considered more amenable to surgical clipping due to their superficial anatomical location and proximity to branch vessels, with excellent outcomes characterized by high rates of complete aneurysm occlusion (>90%) (4). However, in recent years, there has been a growing trend toward EVT for MCA aneurysms (5). In this reported case, the two aneurysms were atypical saccular bifurcation aneurysms; instead, they were large dissecting aneurysms located at the origins of two branches. Direct clipping posed significant challenges in preserving the parent artery, as it risked narrowing the vessel. Moreover, dissecting aneurysms typically lack a true aneurysm neck, rendering clipping ineffective for aneurysm neck repair. Consequently, EVT was selected.

For these two aneurysms, traditional stent-assisted coiling or single-coiling EVT may be technically challenging due to the sharp angle between the MCA trunk and its branches, which complicates stent deployment. Furthermore, since both aneurysms require stent-assisted treatment, deploying double stents within the MCA trunk also presents significant procedural difficulties. For MCA aneurysms, FD deployment can be considered a viable treatment option (6). Therefore, this case selected FD deployment.

Following FD deployment at the MCA bifurcation or trifurcation, a well-documented phenomenon is observed: the covered branch may develop progressive narrowing or occlusion. In Liu et al.'s (2024) meta-analysis of 3,789 patients with intracranial aneurysms treated by FD, the branch narrowing/occlusion rate was 39.2% when FD covered the MCA-M2 segment during a 12-month follow-up (7). Similarly, in Cagnazzo et al.'s (2017) meta-analysis of 244 MCA aneurysms treated with FD, follow-up data revealed that nearly 10% of jailed arteries were occluded, while 26% exhibited slow flow (8).

Based on the phenomenon, FD demonstrates the potential to indirectly treat aneurysms by covering the parent artery origin without aneurysm neck coverage. If the covered branch with the aneurysm is occluded, the aneurysm will be cured. If the covered branch with the aneurysm is narrowed, the aneurysm will be protected from rupture due to decreased blood flow. Multiple mechanisms may contribute to this phenomenon that covered branches by FD may develop narrowing or occlusion (6, 9). Therefore, in this reported case, we aimed to use the FD to treat the two aneurysms by deploying it in the normal middle branch of the left MCA. Fortunately, the expected outcome was achieved. After nearly six months, the two aneurysms were occluded along with the parent arteries, with only one middle branch preserved. The follow-up imaging showing complete aneurysm healing brought the patient great satisfaction.

The mechanism by which the FD narrows or occludes the covered branch is not fully understood. Possible mechanisms include: (1) the flow-diverting effect of the FD, which redirects blood flow from the covered MCA branch into the parent artery; and (2) the straightening of the MCA following FD deployment, which may reduce blood flow to the covered branch. Following the reduction of blood flow to the covered branch by the FD, distal collateral compensation may result in disuse narrowing or even occlusion of the covered branch. Additionally, the FD may induce a neointimal response, thereby increasing the risk of arterial patency compromise and potentially causing branch narrowing and occlusion (6).

Note that the fate of the covered MCA branch remains uncertain after FD deployment. Small branches adjacent to the MCA aneurysm are more likely to occlude, while large branches may face challenges due to the absence of direct collaterals (10). Yavuz et al. (2014) classified MCA occlusion following FD deployment as a predictor of the fate of the covered MCA branch, including: 1) a significant decrease in aneurysm filling (1–3 months); 2) an infundibulum-like appearance due to a branch with a bulking origin caused by aneurysm shrinkage, the so-called “remodeled artery” (3–12 months); and 3) complete occlusion of both the covered branch and the aneurysm (6–18 months) (11). Therefore, FD deployment alone does not cure all MCA aneurysms by merely covering the parent artery origin of the aneurysm. The occlusion of MCA aneurysms may be limited to type 1 and type 2.

In this reported case, apart from the two mechanisms of covered MCA branch occlusion (the flow-diverting effect of the FD and the straightening of the MCA following FD deployment), a third possibility exists: the occlusion in the MCA branches covered by the FD could potentially be attributed to chronic thrombosis. This is because the two MCA branches with dissecting aneurysms exhibited disturbed whirlpool blood flow patterns and increased blood flow resistance, and the distal branches from the aneurysms may have undergone ischemic preconditioning. The FD-induced further reduction of blood flow through coverage of the upper and lower branches of the MCA likely promoted progressive stasis within the aneurysms and their parent arteries, ultimately leading to thrombotic occlusion.

In addition, based on the classification by Yavuz et al., MCA occlusion of both the covered branch and the aneurysm typically occurs 6–18 months after FD deployment (11). In this reported case, the occlusion developed too rapidly, without showing progressive narrowing during the six-month follow-up. However, it was difficult to determine the exact cause of occlusion due to the lack of real-time vascular imaging to monitor changes in the covered branches caused by the FD. Only the final outcome was observed. Because the An FD alone does not provide sufficient dome protection for aneurysms. Adjunctive coiling is often necessary to prevent rebleeding in ruptured aneurysms (12). Dense packing is unnecessary since intra-aneurysmal hemodynamics typically stabilize once the packing density reaches approximately 5% (13). Therefore, in the reported case, loose coiling was employed.

Chronic occlusion of MCA branches covered by FD is generally asymptomatic. According to Cagnazzo et al.'s meta-analysis, FD coverage of MCA branches resulted in symptom rates related to occlusion and slow flow of approximately 5% (8). Liu et al.'s meta-analysis of 3,789 patients with intracranial aneurysms treated by FD covering various branches demonstrated a low incidence rate (<5%) of branch occlusion-related complications, including those involving MCA branches (7). The reported case developed ischemic complications, manifesting as multiple scattered, flaky infarctions. These findings strongly indicate delayed vasospasm as the primary etiology rather than acute branch occlusion, based on: 1) the timing of ischemic events (day 7 post-SAH, corresponding to the peak period for vasospasm), and 2) the characteristic imaging findings of multiple scattered infarctions typical of vasospasm.

For urgent EVT involving FD, DAPT is necessary. Samaniego et al. (2019) reported that the use of the GP IIb/IIIa inhibitor tirofiban and DAPT was safe for ruptured aneurysms, and these agents did not increase the rate of symptomatic hemorrhage complications (14). Therefore, in this present case, tirofiban was used during both the intraoperative and postoperative periods. Due to the high prevalence of clopidogrel non-responsiveness in many Asian patients, and the lack of sufficient time to perform genetic testing for clopidogrel resistance, our institute employed a DAPT regimen of aspirin with ticagrelor. The combined application of DAPT and tirofiban prevented thromboembolic events in this present case.

## 5 Conclusion

This case demonstrates that for mirror-like MCA aneurysms located at the superior and inferior branches of a MCA trifurcation, deploying a FD in the normal branch to cover the origins of the two aneurysmal branches may lead to occlusion of the parent arteries harboring the aneurysms by narrowing or occluding the covered branches. This novel approach offers a potential treatment option.

## 6 Limitation

In this present case, FD deployment to cover the aneurysmal branch origin resulted in occlusion of both the parent artery and the aneurysm, ultimately curing the aneurysm. However, as a case report, these findings are inherently anecdotal, which further limits their generalizability. This method should be cautiously applied as it remains non-routine, and future research with larger cohorts is essential.

## Data Availability

The original contributions presented in the study are included in the article/Supplementary Material, further inquiries can be directed to the corresponding author.
